# A study of tuberculosis in road traffic-killed badgers on the edge of the British bovine TB epidemic area

**DOI:** 10.1038/s41598-018-35652-5

**Published:** 2018-12-06

**Authors:** Elsa Sandoval Barron, Ben Swift, Julian Chantrey, Robert Christley, Richard Gardner, Chris Jewell, Ian McGrath, Andrew Mitchell, Colman O’Cathail, Alison Prosser, Sue Ridout, Gonzalo Sanchez-Cabezudo, Noel Smith, Dorina Timofte, Nicola Williams, Malcolm Bennett

**Affiliations:** 1 School Veterinary Medicine and Science, University of Nottingham, Nottingham, UK; 2 Royal Veterinary College, London, UK; 3 Institutes of Infection and Global Health and Veterinary Science, University of Liverpool, Liverpool, UK; 4 Cheshire Wildlife Trust, Malpas, UK; 5 Lancaster University, Lancaster, UK; 6 Grange Farm, Holmes Chapel, UK; 7 APHA, Weybridge, Addlestone, UK; 8 APHA, Cheshire, UK

## Abstract

The role of badgers in the geographic expansion of the bovine tuberculosis (bTB) epidemic in England is unknown: indeed there have been few published studies of bTB in badgers outside of the Southwest of England where the infection is now endemic in cattle. Cheshire is now on the edge of the expanding area of England in which bTB is considered endemic in cattle. Previous studies, over a decade ago when bovine infection was rare in Cheshire, found no or only few infected badgers in the south eastern area of the county. In this study, carried out in 2014, road-killed badgers were collected through a network of local stakeholders (farmers, veterinarians, wildlife groups, government agencies), and *Mycobacterium bovis* was isolated from 21% (20/94) badger carcasses. Furthermore, there was strong evidence for co-localisation of *M. bovis* SB0129 (genotype 25) infection in both badgers and cattle herds at a county scale. While these findings suggest that both badgers and cattle are part of the same geographically expanding epidemic, the direction of any cross-species transmission and the drivers of this expansion cannot be determined. The study also demonstrated the utility of using road-killed badgers collected by stakeholders as a means of wildlife TB surveillance.

## Introduction

Bovine tuberculosis (bTB) in cattle in Great Britain is concentrated in Southwest England and South Wales, but has been gradually spreading northwards in England^1^. Cattle and people can be infected with several members of the *Mycobacterium tuberculosis* (MTB) complex of closely-related species, but most recent bovine cases in England and Wales have been caused by *M. bovis*. Bovine tuberculosis is of importance both as a zoonosis (although human infection in England and Wales is rare, largely owing to the pasteurisation of milk) and for its effects on international cattle trade and, therefore, the economic and social cost of its control. The epidemiology and control of bTB in cattle is complicated in the UK and the Republic of Ireland by infection in badgers, which can maintain the infection and transmit it to cattle^2,3^. Until recently, there were only sporadic outbreaks of bTB in cattle in Cheshire, in northwest England, one of the more important centres of the British dairy industry. On the basis of both epidemiological investigations of these outbreaks and the genetic analysis (spoligotyping) of *M. bovis* isolates, most such outbreaks were thought to be largely the result of the importation of cattle from endemic areas^4^ further south. Since around 2010, more frequent outbreaks have been reported in herds, particularly in south eastern Cheshire, which by 2013 was on the northernmost edge of the endemic region. The role of badgers in the epidemiology of bTB in Cheshire was, however, unknown. Previous studies in Cheshire, all undertaken more than a decade prior to this study, found no or only few infected badgers, with those few being found in south eastern Cheshire^5,6^.

An important aim of the bTB control strategy in England is to prevent further expansion of the disease in cattle^7^, and it is recognized that this will require much greater understanding of the transmission of *M. bovis* at the geographic edge of the epidemic^7^. While there have been many studies of bTB epidemiology in what are now endemic areas, particularly in southwest England, the role of badgers (and other wildlife) in the northwards geographic expansion of the epidemic in cattle is largely unknown. This expansion could be due to cattle-to-cattle transmission (including over long distances through cattle trading) or due to transmission between badgers, with subsequent cross species transmission. Or the expansion of the epidemic might result from a more complex combination of both processes.

Here we describe a stakeholder-focussed study of *M. bovis* infection in road traffic-killed badgers in and around Cheshire, undertaken between February 2014 and February 2015: the aim of the study was both to determine if bTB could be detected in badgers on the expanding edge of the cattle epidemic and if such an approach might be feasible as a means of wildlife surveillance for bTB. At the time of planning the project, bTB in cattle in much of Cheshire was regarded as sporadic and it was intended that the study investigate bTB in badgers ahead of the epidemic front. In the event, 2014 saw a doubling of recorded bTB outbreaks in Cheshire herds, over a wider area than in previous years, and data from TB surveillance in cattle in Cheshire in 2014 have therefore been included in this study for comparison with the findings in badgers.

## Results

**Figure 1:**
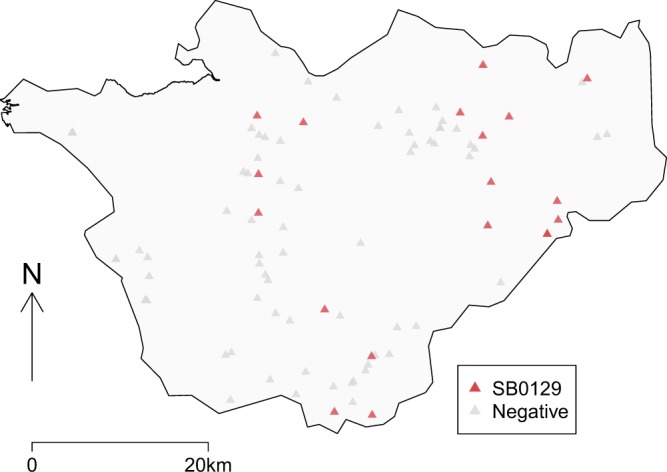
Locations of badger carcasses collected for this study. Culture positive (SB0129) carcasses are shown in red, culture negative carcasses in grey.

Of the 94 badger carcasses collected from within, or very close to, the borders of Cheshire, MTB-complex bacteria were isolated from 20 (Fig. 1, Table 1), all of which were confirmed as *M. bovis* UK spoligotype SB0129 (APHA genotype 25). The full details of all carcasses collected can be found in the supplementary information. Hence, the estimated prevalence of bTB among road killed badgers in this study was 21.3% (CI 95% 14.2 to 30.6%). The prevalence among juvenile badgers was 15.6% (5/32, CI 95% 5.3 to 32.8%) and amongst adults 24.2% (15/62, CI 95% 14.2 to 36.7%). The prevalence among males was 15.4% (8/52, CI 95% 6.9 to 28.1%) and among females 28.6% (12/42, CI 95% 15.7 to 44.6%). The results of a logistic regression (Table 2) showed no evidence of an association between sex and *M. bovis* infection in badgers. Although the number of carcasses collected varied between seasons, with most collected in spring (35% between March and May) and autumn (32% between September and November), compared to winter (16% in February 2014 and December to February 2015) and summer (17% between June and August), the prevalence observed throughout the year was relatively constant with no evidence of seasonal variations (Table 1). No evidence of interaction effects was found between age, sex, or season at the 5% significance level.

**Table 1:** Descriptive statistics for *M. bovis* infection among 94 road-kill badgers collected in Cheshire, UK, over 13 months between 2014 and 2015.

	Number of *M. bovis* culture positive badgers	Total number examined	Prevalence (%)	95% Confidence limits Lower Upper	
Juvenile	5	32	15.6	5.3	32.8
Adult	15	62	24.2	14.2	36.7
Male^†^	8	52	15.4	6.9	28.1
Female	12	42	28.6	15.7	44.6
Winter	4	16	25.0	7.3	52.4
Spring	7	33	21.2	9.0	38.9
Summer	3	15	20.0	4.3	48.1
Autumn	6	30	20.0	7.7	38.6

^†^During post-mortem examination, two pseudohermaphrodite badgers were identified, one juvenile and one adult. For further analyses these two badgers were considered as males as they were most likely male pseudohermaphrodites according to Bigliardi *et al*.^50^.

**Table 2:** Results of logistic regression for TB positivity in badgers against age, sex, and season.

		Odds Ratio	p value	Std Error
Intercept (adult, female, autumn)		0.584	0.371	0.601
Young		0.429	0.168	0.614
Male		0.370	0.066	0.542
Season	Spring	0.839	0.789	0.657
	Summer	0.906	0.903	0.808
	Winter	1.410	0.651	0.759

The Intercept represents the baseline odds of being TB positive for an adult, female badger collected in autumn.

Only one badger had characteristic, visible TB lesions, which were found in widespread lymph nodes and in other organs, including lung, liver, spleen and kidney. This badger was an adult and, based on body mass and dental wear, was estimated to be over 3 years old. Seven other badgers (four culture-positive and three culture-negative) had similar age-related dental wear. Smears from visible lesions stained positive for acid-fast bacteria, and histological examination of fixed lung lesions demonstrated characteristic necrogranulomas containing numerous acid-fast bacilli. All typical tuberculous lesions yielded *M. bovis* in culture.

As in previous studies^8,9^, lymph nodes and other sampled tissues were pooled for culture. *M. bovis* was cultured from 36 tissue pools from the 20 TB-positive badgers. The tissue pools most frequently positive were those comprising the ‘carcass’ lymph nodes (i.e. pools of axillary, prescapular, and inguinal lymph nodes), and the pools of head and neck lymph nodes. Each of these pools was culture positive for 12/20 positive badgers. Thoracic lymph nodes and abdominal pools (mesenteric and hepatic lymph nodes, liver, kidney and spleen) were positive from 6 and 5 carcasses, respectively. Only the carcass with visible lesions, including in the lung, had a culture positive lung pool. Of the 20 culture positive carcasses, 10 had only one positive tissue pool, 8 between 2 and 3 pools and only 2 carcasses had 4 or more culture-positive tissue pools.

**Figure 2:**
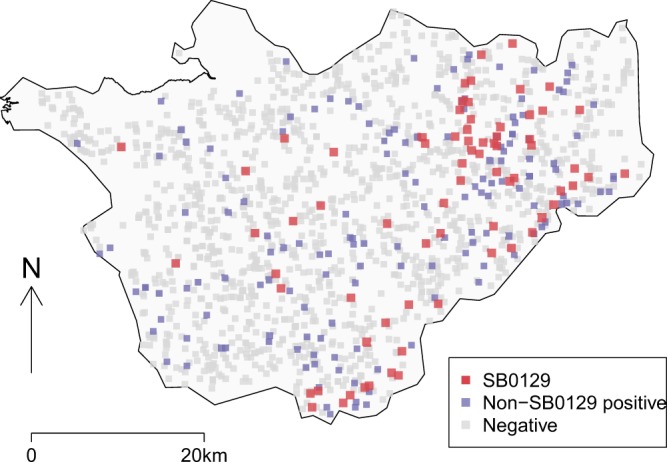
Locations of cattle farms and holdings in Cheshire. Negative holdings are shown in grey, positive SB0129 holdings in red and positive non-SB0129 holding in blue.

The results of cattle TB testing in Cheshire over the same period as the badger study were compared with the results of testing badgers. In total, there were 2611 herd-level tests at 1552 cattle holdings in Cheshire in 2014: the median number of tests/holding was 1, with a range of 1–7 tests/holding (Fig. 2). More than two herd-level tests per holding was largely associated with the detection of infection (test positive cattle were removed and herds retested until negative), and the number of routine surveillance tests was 1758 (mean 1.13 test/holding, range 1–3). Reported herd sizes ranged from 1 to 2569 animals (median 82, IQR 20-231). There were 197 herd ‘breakdowns’ (13% of holdings), i.e. at least one animal tested positive on at least one occasion in 2014. Of these, 29 herds had also tested positive the previous year.

The predominant spoligotype found in cattle during 2014 was SB0129 (genotype 25), particularly in the east of Cheshire (Fig. 2)^10^. *M. bovis* isolates from cattle in other areas were more often other spoligotypes, prevalent in other regions of England, and frequently confirmed by epidemiological tracing to be associated with cattle movements into Cheshire.

**Figure 3:**
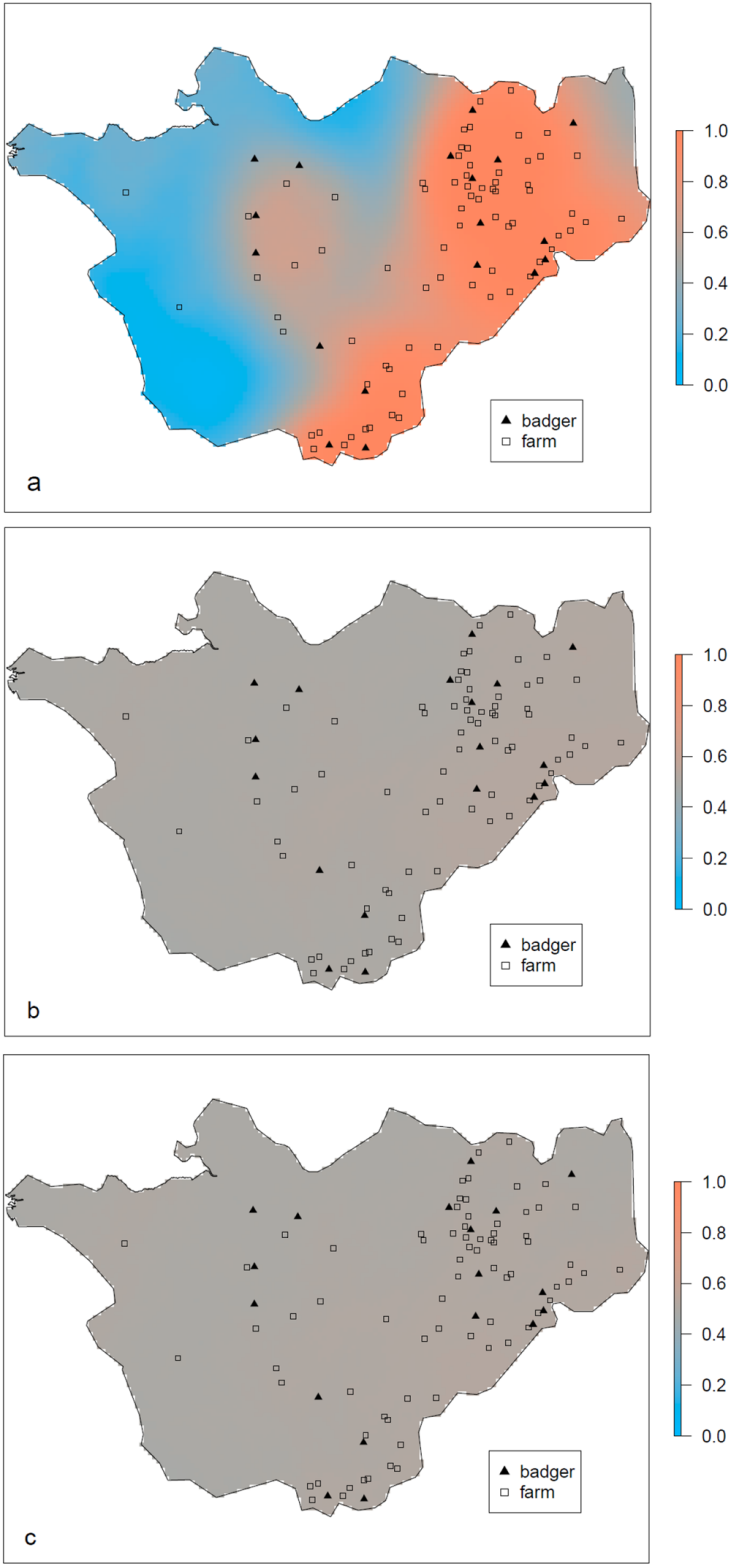
Spatial exceedance maps. Spatial exceedance (Pr(S_z_(x_i_) > 0), z = {c, b, f}) maps for residual bTB SB0129 infection risk: combined spatial risk (**a**), badger excess risk (**b**), farm excess risk (**c**).

Spatially co-incident bTB prevalence between badgers and cattle farms was examined using a ‘linear model of co-regionalisation’ technique^11^. Prevalence at each observation point was adjusted for observation type (i.e. badger or farm), with spatial excess risk surfaces allowing for spatially-varying risk common to both badgers and farms, and additional spatially-varying risk for badgers and farms separately. The results were summarised as exceedance probability maps, showing the probability of the spatial odds ratio for SB0129 infection exceeding 1. At any location within the study region, values of 0.5 indicate equal probability that the odds ratio for infection exceeds or is less than 1, in other words that there is no evidence of excess risk at that location. Conversely, departures from 0.5 indicate evidence of spatially-varying disease risk. Figure 3 shows a marked spatial variation in disease prevalence irrespective of being a badger or a farm (Fig. 3a), whereas no evidence was found of additional spatial variation in prevalence for badgers (Fig. 3b) and farms (Fig. 3c) separately. Taken together, these results show no evidence for differing spatial distributions of *M. bovis* spoligotype SB0129 infections in either badger populations or cattle herds, indicative of co-localisation of *M. bovis* SB0129 infection in both badger populations and cattle herds at a county scale.

## Discussion

This study demonstrated the utility of a stakeholder-focussed approach, using road-killed badgers collected by stakeholders as a means of wildlife TB surveillance. Overall, stakeholders collected, in just over 12 months, 94 carcasses from Cheshire. *M. bovis* was isolated from 21% badger carcasses and, importantly, there was strong evidence for co-localisation of *M. bovis* SB0129 (genotype 25) infection in both badger populations and cattle herds at a county scale. However, this study could not determine the direction(s) of transmission between the two species. Whole genome sequencing and comparison of geographically-related isolates from cattle and badgers might enable better understanding of any inter-species transmission. These findings accord with those of Goodchild *et al*.^9^ in Wales and Abernethy *et al*.^12^ in Northern Ireland, who also found an association between the infection status of dead badgers and presence of cattle TB incidents within a 3–5 km radius.

The overall prevalence of *M. bovis* isolated from road-killed badgers in the Cheshire area, 21% (CI 95% 14.2 to 30.6), is similar to the prevalence estimates of 18% and 20% in Worcestershire and Gloucestershire respectively^13^ in southwest England, both ‘high-risk’ counties for cattle infection. The 2014 prevalence was much higher than that found in road-killed badger surveys in Cheshire between 1972 and 1990 when only one positive badger out of 389 collected (0.26%) was found^5^. Although highly specific, the sensitivity of culture for diagnosing *M. bovis* infection in badgers is likely to mean the calculated prevalence is underestimated.

Previous studies found that the prevalence of infection in badgers was higher in adults than cubs^14^, and in males than females^13,15–17^, although in most cases these differences were not statistically significant. This study also found no significant difference in prevalence by sex or age.

The majority of positive cultures were from the ‘head and neck’ and ‘carcass’ pools of lymph nodes (33% for each). This is in contrast to most previous studies, which found that isolation of *M. bovis* was most frequent from lung and thoracic lymph nodes, leading to the suggestion that tuberculosis in badgers is primarily a respiratory disease resulting from aerosol infection^8,18^. However, Corner *et al*.^19^ also found a high frequency of infection of axillary lymph nodes, and suggested that isolation from peripheral lymph nodes could be due to extra-pulmonary dissemination of infection or a secondary pathogenic pathway through the mucosa of the upper respiratory tract. Although some authors have reported bite wounds and external lesions as common and as possible sources of *M. bovis*^20–22^, no badger carcasses in this study had obvious bite wounds. However, it is possible that the carcass damage caused by vehicular collision or post-mortem scavengers masked their presence.

Road traffic and ‘found dead’ surveys have previously been used to study several wildlife diseases^23–29^, including bTB in badgers^6,9,12^. Such surveys can be influenced by a range of spatial and temporal factors that include: animal density and behaviours, road type and use, seasonality, collection convenience and safety, and engagement by those reporting or collecting carcasses^25,30^. It is likely that similar biases may apply to this study. For example, there was a possible concentration of carcasses submitted from areas with bTB outbreaks in cattle, and this might reflect the majority of carcasses having been submitted by farmers, some of whom might in turn have a particular interest in having local badgers tested. Larger scale studies would be needed to examine such potential biases, and also any relationship between badger carcasses submitted with types of road (in this study it was not possible to sample badgers from major roads on which stopping is illegal), local badger population size and ranges, type of landscape, and other factors affecting local stakeholder engagement. In future studies of road killed badgers, ensuring a wider range of stakeholders actively engage in carcass collection might mitigate some potential biases in the spatial distribution of sampled carcasses.

There are no county-wide surveys of badgers in Cheshire with which to compare the distribution of carcasses collected in this study. Unpublished data collected by Cheshire Wildlife Trust during its TB badger vaccination campaign in 2013 and 2014 found estimated densities of up to 10.5 badgers per km^2^. These estimates may be inflated by their being based on surveys of areas with known badger populations, but suggest densities in some areas of Cheshire not dissimilar to those found in the South-West areas of England in 2010, where densities ranged from 1.5–4.8 per km^2 ^^31^.

Although there is some evidence that bTB can affect badger behaviour^20^, which might influence the risk of badgers being hit by vehicles, other studies have suggested that ‘latent’ bTB (which predominated in our study) has minimal effect on the life histories of badgers^32,33^. Hence, it is unlikely that bTB infection would have caused sampling bias in this study. This is further supported by the finding that key demographic parameters, including the ratio of males to females (approximately 1:1) and of adults to juveniles (2:1) was similar to previous studies, whether based on trapping live badgers or road-killed badgers^16,23,34–36^.

In conclusion, this study demonstrated a relatively high prevalence of *M. bovis* infection in badgers in Cheshire, a county on the edge of the bTB epidemic in cattle in England, and no significant differences between the spatial distribution of cattle TB outbreaks and infected badgers. It also demonstrated that stakeholder engagement in road traffic-killed surveys can be a useful approach to surveillance of bTB in wildlife. The findings suggest a marked increase in bTB in road-killed badgers in Cheshire over 25 years, not dissimilar to the increased incidence of cattle disease in the same area and over the same time. However, they do not address the question as to which population drives the expanding edge of the national bTB epidemic. Spoligotyping of both badger and cattle isolates suggest that the main Cheshire epidemic (due to *M. bovis* SB0129) is an expansion from counties to the south and east, and not the result of distantly imported spoligotypes, which remain responsible for continuing sporadic cattle outbreaks in west and north Cheshire. Further studies, involving comparison of whole genome sequences of 2014 isolates from both cattle and badgers, might provide a better understanding of ‘who infects whom’.

## Methods

### Badgers and sample collection

The study was developed with and through Cheshire TB Eradication Group, an informal body comprising farmers, veterinarians (both in private practice and working for the Animal and Plant Health Agency, APHA), Cheshire Wildlife Trust and local badger protection groups, the National Farmers Union (NFU) and other stakeholders including Local Authorities and livestock markets. The group met, and continues to meet, regularly in order to share information and experiences, and to discuss bTB and its control in Cheshire.

Members of the group collected fresh badger carcasses seen on roads while travelling as part of their normal daily activities within the study area. An additional five carcasses were donated by the Royal Society for the Prevention of Cruelty of Animals (RSPCA) or local veterinary surgeons after they had to be euthanized or died after admission to their medical centres. Only badgers collected within or very close to the traditional borders of Cheshire were included in this study. Those collecting carcasses were given ‘collection kits’ that included gloves, masks, sealable transport bags, submission forms and guidance on how to collect and transport the carcasses safely. The group was also given safety advice as part of regular briefings. The risk assessments and guidance were all approved through the University of Liverpool’s safety processes. Ethical approval was also provided through the University of Liverpool.

Carcasses estimated at being no more than two days dead, were delivered to the University of Liverpool’s School of Veterinary Science where, after storage at 4 °C for no more than 24 hours, standardised post mortem examination and tissue sampling were undertaken inside a Containment Level 3 (CL3) laboratory. Carcasses with autolytic changes suggesting death more than three days prior to necropsy, or with an open abdomen through trauma and so likely to be heavily contaminated, were not included in the study. Furthermore, with only a few exceptions (e.g. carcasses from wildlife rescue organisations), only carcasses clearly involved in road traffic accidents were accepted into the study. As badgers are protected by UK law, contributors were warned that any evidence of illegal trapping or killing would be reported to the police: no such evidence was seen. The age of animals was estimated from dentition, body mass and size and recorded as juvenile (<1 year) or adult (>1 year)^37^.

Samples taken during the post-mortem examination included: any visible lesions compatible with TB in any organ, superficial, thoracic and abdominal lymph nodes, lung lobes, spleen, liver and kidneys. For culture, each lesion was processed separately but tissue pools were created from non-lesioned material: a ‘lung pool’ of lung lobe samples, a ‘thoracic pool’ of bronchial and mediastinal lymph nodes, an ‘abdominal pool’ comprising liver, spleen, kidneys and hepatic and mesenteric lymph nodes, a ‘head and neck’ pool of parotid, mandibular, retropharyngeal and cervical lymph nodes, and a ‘carcass pool’ of prescapular, axillary, and superficial inguinal lymph nodes. Smears of lesion material were stained for acid-fast bacteria using a modified Ziehl-Neelsen method^38^, and tissues with visible lesions were fixed in 10% formol saline and examined histopathologically. Individual tissue samples were stored at −80 °C in case needed for further study.

### Isolation of *M. bovis* from badgers

All tissue processing and culture was undertaken in a CL3 facility. Tissue pools were gently ground with sterile sand and 2–3 ml of phosphate buffer solution, then mixed with an equal amount of 5% oxalic acid for 5 to 10 min to reduce contamination with bacteria other than mycobacteria^39,40^. Pooled samples were inoculated on to both Stonebrink Selective agar (BD Diagnostics, Oxford) and Lowenstein-Jensen with pyruvate (Media for Mycobacteria Ltd. Penarth) slopes and incubated at 37 °C for a minimum of 12 weeks^41^. Cultures were examined weekly for the appearance of colonies characteristic of *M. bovis*.

### Characterisation of *M. bovis* isolates from badgers

DNA was extracted from presumptive *M. bovis* colonies by heating a suspension of 1–2 colonies in 100 µl DNA-free water at 80 °C for 30 min and/or by use of Qiagen DNA extraction kits using the manufacturer’s instructions. Isolates were confirmed as MTB complex by PCR amplification of the IS6110 genetic element^42^. Spoligotyping was carried out at APHA, Weybridge^43^, as well as at the University of Nottingham using DNA microarray technology (Alere Technologies, Germany). Briefly, extracted DNA from colonies was diluted 1:1000 into sterile distilled water. This was used as template DNA to amplify and, using biotinylated primers, label the DR region of the *M. bovis* genome^44^. The amplified DNA was then hybridized onto ArrayStrips using the *M. bovis* spoligotyping array kit (Alere) according to Ruettger *et al*.^44^. In addition, nine isolates had their spoligotypes confirmed by inference from their whole genome sequences. Spoligotypes were described using the international designation (MBovis.org)^45^.

### Cattle data

Cattle testing data for Cheshire were obtained from APHA’s surveillance programme. During 2014, cattle in Cheshire were tested at herd level annually, with additional herd-level tests after 60 days if any cattle tested positive or if neighbouring (within 3 km) cattle holdings tested positive for bTB^10^. Furthermore, some holdings had groups of cattle tested before or after movements, or because of epidemiological tracing of outbreaks elsewhere in the country. Some positive cattle were also detected during routine inspection in the abattoir. The original data sets from APHA included cattle holding location, the results of each test (including spoligotype if known), the official ‘breakdown identifier’ and start date, and the numbers of cattle on the holding and tested on each occasion. For the purposes of this study, all test results in 2014 were combined for each holding, and TB-positive herds were defined as those with at least one animal having tested positive using the ‘single intradermal comparative cervical tuberculin’ (SICCT) test, at least one animal with two consecutive inconclusive skin test results, positive interferon gamma tests, or if lesions which were culture positive were found at the abattoir^46^. Spoligotyping of cattle isolates was as described above^43^: only one isolate per outbreak was spoligotyped.

### Statistical analyses

Prevalence determination was not the intended aim of this study as the overall population of badgers in Cheshire was unknown, but known to vary greatly across the area (Cheshire Wildlife Trust, unpublished data). However, the prevalence was estimated among the sampled badgers, as in previous studies^9,12^, on the assumption that the carcasses collected were representative of the overall population.

Descriptive statistics for prevalence by age, sex, and season of submission were calculated using the exact binomial method. A multivariable logistic regression was used to detect associations between age, sex and season of submitted carcasses with bTB infection status. The effect of including two-way interaction terms between all three explanatory variables were tested sequentially using likelihood ratio tests.

To detect evidence for co-localisation of SB0129 *M. bovis* positive badgers and SB0129 herd breakdowns, Bayesian Bernoulli co-regionalisation geostatistical models were used^11,47^. In this case, the observations consisted of a set of geographically located farms and badgers, with infection status as an outcome variable (positive or negative), and observation type (farm or badger) as an explanatory variable. The outcome was modelled for each observation, *y*_*i*_, as a Bernouilli random variable with probability *p*_*i*_ such that

$${\rm{logit}}({p}_{i})=\{\begin{array}{c}\alpha +\beta +{S}_{c}({x}_{i})+{S}_{b}({x}_{i})\,{\rm{if}}\,i\,{\rm{is}}\,{\rm{a}}\,{\rm{badger}}\\ \alpha +{S}_{c}({x}_{i})+{S}_{f}({x}_{i})\,{\rm{if}}\,i\,{\rm{is}}\,{\rm{a}}\,{\rm{farm}}\end{array}$$

where *x*_*i*_ is the geographical location of observation *I*, and *α* and *β* represent respectively the intercept and the log odds ratio for disease in badgers versus farms. The model makes use of three spatial Gaussian Processes, ${S}_{c}({x}_{i})$, ${S}_{b}({x}_{i})$, and ${S}_{f}({x}_{i})$, characterised by a covariance function ${\Sigma }_{ijz}$ between the locations of observations I and j for each of the Gaussian processes $z=\{c,b,f\}$. In each case, we assume a Matérn correlation structure such that

$${\Sigma }_{ijz}={\sigma }_{z}(1+\frac{\surd \parallel 3{x}_{i}-{x}_{j}\parallel }{{\varphi }_{z}})\exp (-\frac{\surd 3\parallel {x}_{i}-{x}_{j}\parallel }{{\varphi }_{z}})$$

with sill and scale parameters ${\sigma }_{z}$ and ${\varphi }_{z}$^48^. Here, ${S}_{c}({x}_{i})$ represents a disease risk surface common to both farms and badgers that allows disease prevalence to vary with space. Then, ${S}_{b}({x}_{i})$ and ${S}_{f}({x}_{i})$ represent respectively the *additional* spatial risk for badgers and farms respectively. In a Bayesian context, prior distributions were assigned to the parameters as shown in Table 2. The model was implemented in Python 2.7 using the PyMC3 module^48^, with the No-U-Turn Sampler (NUTS) used to draw samples from the joint posterior distribution. The sampler was first initialised using 4000 iterations of variational inference, followed by a pilot run of 1500 NUTS iterations, before a ‘production’ run of 3000 samples was obtained for inference purposes.

To detect co-regionalisation of SB0129 in both badgers and farms, the Bayesian predictive distribution of the Gaussian Process was evaluated on a fine grid over the Cheshire study area^49^. To summarise each predictive distribution, the probability that the Gaussian Process exceeded 0 (i.e. no spatial effect) at each grid point – the exceedance probability – was calculated and plotted as a map. In a Bayesian context, evidence for a spatial effect is shown as the exceedance probability being different from 0.5 (i.e. equal probability of the spatial prevalence being either low or high compared to the overall mean prevalence).

## Electronic supplementary material

Badger carcasses supplementary data
