# Supplementary material for: A study of tuberculosis in road traffic-killed badgers on the edge of the British bovine TB epidemic area
Source: Sci Rep. 2018 Dec 6;8:17206. doi: 10.1038/s41598-018-35652-5 (PMC6283848; doi:10.1038/s41598-018-35652-5)
Supplement: Supplementary file 1 — Badger carcasses supplementary data [file 41598_2018_35652_MOESM1_ESM.pdf]

A study of tuberculosis in road traffic-killed badgers on the edge of the British bovine TB epidemic area

Elsa Sandoval Barron[1]*, Ben Swift[2], Julian Chantrey[3], Robert Christley[3], Richard Gardner[4], Chris Jewell[5], Ian McGrath[6], Andrew Mitchell[7], Colman O'Cathail[1],

Alison Prosser[7], Sue Ridout[8], Gonzalo Sanchez-Cabezudo[8], Noel Smith[7], Dorina Timofte[3], Nicola Williams[3], Malcolm Bennett[1]*

School Veterinary Medicine and Science, University of Nottingham[1], Royal Veterinary College[2],Institutes of Infection and Global Health and Veterinary Science, University of Liverpool[3], Cheshire Wildlife Trust[4], Lancaster University[5], Grange Farm[6], APHA, Weybridge[7], APHA Northern Region[8]

BADGER ID	DATE OF SUBMISSION	LOCATION		SEX	AGE GROUP	WEIGHT	bTB	SP	APHA genotype	CULTURE POSITIVE POOLS					
		LATITUDE	LONGITUDE							Head	Thorax	Lungs	Abdomen	Carcass	Other
1	13/02/2014	53.185588	-2.156694	F	A	10.7	+	SB0129	25		+				
2	17/02/2014	52.998867	-2.503158	M	A	11									
3	18/02/2014	53.122994	-2.905080	M	A	9.5									
4	20/02/2014	52.986658	-2.597711	F	Y	9.6									
5	25/02/2014	53.001451	-2.641757	M	A	9.8									
6	22/02/2014	53.197293	-2.596685	F	Y	7.9									
7	24/02/2014	53.081231	-2.853130	M	A	11									
8	24/02/2014	53.081059	-2.851652	M	Y	11									
9	26/02/2014	53.310682	-2.105841	M	Y	10.5	+	SB0129	25					+	
10	27/02/2014	53.306314	-2.581477	M	Y	11									
11	04/03/2014	52.994913	-2.534447	M	A	11.5									
12	04/03/2014	53.009849	-2.581742	O	A	11									
13	04/03/2014	53.234952	-2.406369	M	A	11									
14	06/03/2014	53.306667	-2.114735	F	A	11									
15	10/03/2014	53.081153	-2.855131	F	A	10									
16	10/03/2014	53.324072	-2.283821	F	A	12	+	SB0129	25		+				
17	16/03/2014	53.073623	-2.550301	F	Y	8.5	+	SB0129	25				+		
18	24/03/2014	53.264487	-2.589037	M	A	10	+	SB0129	25		+				
19	27/03/2014	53.084625	-2.664641	M	A	10									
20	27/03/2014	53.131213	-2.621562	M	Y	9.4									
21	27/03/2014	53.011436	-2.480388	F	Y	9.5									
22	27/03/2014	52.966901	-2.706037	F	A	12									
24	01/04/2014	52.966329	-2.468750	F	A	11	+	SB0129	25					+	
25	02/04/2014	53.213448	-2.690132	M	A	11.5									
26	10/04/2014	53.173164	-2.718004	M	Y	7									
28	14/04/2014	53.254551	-2.408571	M	A	8.5									

29	16/04/2014	53.026211	-2.470341	F	A	10	+	SB0129	25	+	+				
30	16/04/2014	53.166378	-2.154942	F	A	8	+	SB0129	25					+	
31	17/04/2014	53.238635	-2.297403	M	Y	11									
32	21/04/2014	53.067185	-2.524102	F	Y	4.5									
33	28/04/2014	53.258027	-2.677223	F	A	9									
34	29/04/2014	53.026195	-2.717450	M	A	11									
35	30/04/2014	53.000401	-2.500750	F	A	9									
36	30/04/2014	53.128161	-2.662534	M	A	11									
37	30/04/2014	53.289743	-2.532730	F	A	8.5									
39	02/05/2014	53.323941	-2.281935	F	Y	11									
40	06/05/2014	53.277426	-2.426571	F	A	11									
41	07/05/2014	53.227583	-2.666000	M	A	7.8									
42	09/05/2014	53.055745	-2.427419	M	A	10									
43	15/05/2014	53.164097	-2.675660	M	A	12									
45	27/05/2014	53.266756	-2.352166	F	A	9.5									
46	28/05/2014	53.211959	-2.682786	M	A	9									
47	31/05/2014	52.969168	-2.532553	M	A	7	+	SB0129	25	+	+	+	+	+	
48	11/06/2014	53.124957	-2.851486	F	Y	10									
49	12/06/2014	53.171628	-2.664184	F	Y	8	+	SB0129	25	+					
51	16/06/2014	53.259430	-2.356407	F	A	11									
52	16/06/2014	53.258789	-2.328058	F	A	8.5									
53	23/06/2014	53.204119	-2.627492	M	A	9.5									
54	27/06/2014	53.108519	-2.651180	F	Y	7.5									
55	03/07/2014	53.119771	-2.661633	F	Y	6									
56	05/07/2014	53.028846	-2.707823	M	A	8									
57	08/07/2014	53.157084	-2.621773	M	A	4.5									
58	03/08/2014	52.980492	-2.708575	M	A	9									
59	06/08/2014	53.259946	-2.356157	M	Y	11.5									
60	07/08/2014	53.122631	-2.905024	M	A	10									

61	08/08/2014	53.151901	-2.173711	M	A	4	+	SB0129	25	+	+		+	+	
62	16/08/2014	53.151828	-2.173262	F	A	9.5	+	SB0129	25	+			+	+	
63	23/08/2014	53.028386	-2.440336	M	A	12									
64	01/09/2014	53.253709	-2.072184	F	Y	7.5									
65	03/09/2014	53.241766	-2.399562	F	A	11									
66	03/09/2014	53.160318	-2.274535	M	Y	9.5									
67	07/09/2014	53.160318	-2.274535	F	A	10	+	SB0129	25	+				+	
68	09/09/2014	53.251177	-2.982189	M	Y	10.5									
69	09/09/2014	52.979292	-2.501709	F	Y	11									
70	11/09/2014	53.103036	-2.646691	F	Y	7									
71	11/09/2014	53.141542	-2.490284	F	Y	8									
72	12/09/2014	53.057124	-2.395268	F	Y	8									
73	18/09/2014	53.102020	-2.252361	F	A	9									
74	19/09/2014	53.242498	-2.304376	M	A	13									
75	19/09/2014	53.204796	-2.269233	F	Y	12	+	SB0129	25	+				+	
76	23/09/2014	53.252049	-2.982099	F	A	9									
77	24/09/2014	53.027086	-2.464493	M	A	10									
78	25/09/2014	53.243637	-2.365923	F	Y	14.5									
79	26/09/2014	53.159816	-2.094279	M	A	13	+	SB0129	25	+				+	
80	02/10/2014	53.246260	-2.372866	M	A	16									
81	02/10/2014	53.211190	-2.664720	M	A	9.5	+	SB0129	25	+				+	
82	12/10/2014	53.334875	-2.637863	M	A	13.5									
83	13/10/2014	53.131942	-2.865458	M	A	13									
84	22/10/2014	53.280585	-2.363183	M	A	18									
85	24/10/2014	53.245164	-2.627852	O	Y	13.5									
86	28/10/2014	53.251863	-2.283800	M	Y	9.5	+	SB0129	25				+		
87	06/11/2014	53.017480	-2.476240	M	Y	13.5									
88	11/11/2014	53.261209	-2.461615	M	A	12									
89	11/11/2014	53.251221	-2.664148	M	A	14									

90	13/11/2014	53.271591	-2.238900	F	A	15	+	SB0129	25	+				+	
91	14/11/2014	53.061900	-2.609300	F	Y	11									
92	18/11/2014	53.230752	-2.305848	M	A	13.5									
93	19/11/2014	53.249013	-2.654189	M	A	15									
95	09/12/2014	53.250900	-2.088400	F	A	6									
96	09/12/2014	53.275500	-2.322200	F	A	14	+	SB0129	25	+				+	
97	20/01/2015	53.069212	-2.633437	M	Y	12.5									
98	20/01/2015	53.105857	-2.848439	F	Y	8.5									
99	23/01/2015	53.018888	-2.367947	M	A	10									
102	29/01/2015	53.270986	-2.667939	M	A	12.5	+	SB0129	25	+					

Sex was classified as Male (M), Female (F) or Other (O). All carcasses in the O category were considered males

Age was classified as Adult (A) over a year old and Juvenile (Y) under a year old
